# Early changes to retinal structure in patients with diabetic retinopathy as determined by ultrawide swept-source optical coherence tomography-angiography

**DOI:** 10.3389/fendo.2023.1143535

**Published:** 2023-05-08

**Authors:** Yong Zeng, Miao Liu, Mengyu Li, Dinyang Wei, Mingzhu Mao, Xinyue Liu, Sizhu Chen, Yang Liu, Bo Chen, Lei Yang, Sanmei Liu, Lifeng Qiao, Ruifan Zhang, Jie Li, Wentao Dong, Jie Zhong

**Affiliations:** ^1^ Department of Ophthalmology, Sichuan Provincial People’s Hospital, University of Electronic Science and Technology of China, Chengdu, China; ^2^ Department of Ophthalmology, Chinese Academy of Sciences Sichuan Translational Medicine Research Hospital, Chengdu, China; ^3^ Jinniu Maternity and Child Health Hospital of Chengdu, Department of Child Healthcare, Chengdu, China; ^4^ Department of Pulmonary and Critical Care Medicine, Enyang District People’s Hospital of Bazhong, Bazhong, Sichuan, China

**Keywords:** vascular density (VD), thickness, diabetes mellitus, diabetic retinopathy, SS-OCTA, peripheral retina

## Abstract

**Purpose:**

To investigate retinal vascular changes in patients with diabetic retinopathy (DR) using the newly developed ultrawide rapid scanning swept-source optical coherence tomography angiography (SS-OCTA) device.

**Methods:**

This cross-sectional, observational study enrolled 24 patients (47 eyes) with DR, 45 patients (87 eyes) with diabetes mellitus (DM) without DR, and 36 control subjects (71 eyes). All subjects underwent 24 × 20 mm SS-OCTA examination. Vascular density (VD) and the thickness of the central macula (CM; 1 mm diameter) and temporal fan-shaped areas of 1–3 mm (T3), 3–6 mm (T6), 6–11 mm (T11), 11–16 mm (T16), and 16–21 mm (T21) were compared among groups. The VD and the thicknesses of the superficial vascular complex (SVC) and deep vascular complex (DVC) were analyzed separately. The predictive values of VD and thickness changes in DM and DR patients were evaluated by receiver operating characteristic (ROC) curve analysis.

**Results:**

The average VDs of the SVC in the CM and the T3, T6, T11, T16, and T21 areas were significantly lower in the DR than in the control group, whereas only the average VD of the SVC in the T21 area was significantly lower in the DM group. The average VD of the DVC in the CM was significantly increased in the DR group, whereas the average VDs of the DVC in the CM and T21 area were significantly decreased in the DM group. Evaluation of the DR group showed significant increases in the thicknesses of SVC-nourishing segments in the CM and T3, T6, and T11 areas and significant increases in the thicknesses of DVC-nourishing segments in the CM and T3 and T6 areas. In contrast, none of these parameters showed significant changes in the DM group. ROC curve analysis showed that the average VD of the SVC in the CM, T3, and T21 had better ability to predict DR, with areas under the ROC curves (AUCs) of 0.8608, 0.8505, and 0.8353, respectively. The average VD of the DVC in the CM was also predictive of DR, with an AUC of 0.8407.

**Conclusions:**

The newly developed ultrawide SS-OCTA device was better able to reveal early peripheral retinal vascular changes than traditional devices.

## 1 Introduction

Diabetes mellitus (DM) is a common and frequently occurring disease worldwide that was estimated to affect 536.6 million adults aged 20-79 years in 2021 (1). Diabetic retinopathy (DR) is a frequent ocular complication of DM, characterized by neurovascular injury and vision impairment (2). Early screening and therapies for DR are now considered effective for preventing blindness or vision loss (3). Although patients have been screened for DR using dilated and seven-field fundus photography techniques (4, 5), these methods cannot analyze blood flow or deep retinal changes. Microaneurysms, venous beading, vascular leakage, and nonperfused areas can be visualized by fundus fluorescein angiography, but this method is invasive, time-consuming, and not quantitative, and is associated with a risk of allergy (6, 7). Optical coherence tomography (OCT) is a type of ocular fundus examination that can obtain information on retinal layers *via* tomography scans. This method is widely used in the diagnosis and follow-up of patients with diabetic macular edema and other macular diseases (8–10), although it cannot determine blood flow.

**Figure 1:**
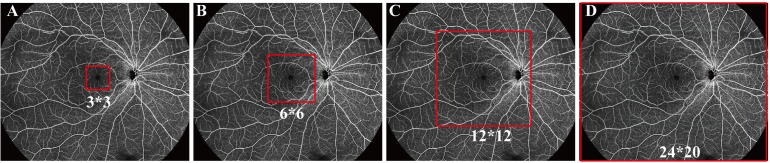
Fields detected by optical coherence tomography angiography (OCTA). **(A)** 3 × 3 mm, **(B)** 6 × 6 mm, **(C)** 12 × 12 mm, and **(D)** 24 × 20 mm.

OCT angiography (OCTA) is a rapid, noninvasive, and high-resolution ocular fundus examination that can simultaneously perform tomography scans and obtain blood flow information (11, 12). The high resolution of OCTA can determine the occurrence of preclinical retinopathy, by, for example, identifying changes in the foveal avascular zone, as well as alterations in vessel density and retinal thickness (11, 13). Although imaging by OCTA was previously limited to a 3 × 3 mm or 6 × 6 mm area in the center of the macula, this method can acquire high-resolution tomography and angiography images of 12 × 12 mm and even 24 × 20 mm areas of the retina and choroid using a single capture (**Figure 1**) (14, 15). These types of wide-field OCTA can provide considerable information on the retina and choroid, broadening knowledge of DR and other ocular fundus diseases.

The TowardPi swept-source (SS) OCT and OCTA system (BMizar, TowardPi Medical Technology, Beijing, China) is a newly developed high-resolution and wide-field tomographic and angiography imaging system with an A-scan rate as high as 400 KHz and an axial scan depth of 6 mm. This system can acquire 24-mm-wide tomography and 24 × 20 mm angiography information in about 15 seconds in a single capture (16). The present study utilized this system to analyze baseline parameters in patients with DR, patients with DM without DR, and normal controls, including vascular density and thickness of various areas of the superficial and deep layers of the retina. Use of this system may help determine changes in the peripheral retina of patients with DR, suggesting its usefulness in early screening for this condition.

## 2 Materials and methods

### 2.1 Participants

This cross-sectional, observational study enrolled 24 patients (47 eyes) with DR, 45 patients (87 eyes) with DM without DR, and 36 control subjects (71 eyes) who visited the Department of Ophthalmology at Sichuan Provincial People’s Hospital in Chengdu, Sichuan, China between January and July 2022. The study was conducted in accordance with the Declaration of Helsinki and was approved by the Ethics Committee of Sichuan Provincial People’s Hospital (permit number 2022–258). All participants provided written informed consent prior to enrollment.

### 2.2 Inclusion and exclusion criteria

DR was diagnosed according to the criteria Early Treatment Diabetic Retinopathy Study. DM was diagnosed according to the criteria set by the World Health Organization (WHO) (17). Patients were included if they (1) were aged >18 years, (2) had been definitively diagnosed with type 2 DM, (3) did not have an unknown cause of vision loss, and (4) had an OCTA image quality index >8. Patients were excluded if they had (1) severe opacity of the cornea, lens, or vitreous; (2) glaucoma, high myopia, or other ocular diseases that might influence blood flow in the retina; (3) a history of nondiabetic chorioretinopathy or ocular fundus surgery or photocoagulation; or (4) insufficient medical data (e.g., DM history, current treatment protocol, or glycemic control). The participants were then divided into the DR, DM, and normal control (NC) groups based on their diagnosis.

### 2.3 Demographic data and ophthalmic examinations

Demographic and clinical characteristics of all subjects were recorded, including their age, gender, height, weight, past medical history, duration of diabetes, type of diabetes, and treatment. All subjects underwent ophthalmic examinations, including optometry, and measurements of intraocular pressure (IOP) and axial eye length (AL). Other factors recorded included best corrected visual acuity (BCVA) and refractive error. Anterior segments were examined by slit-lamp biomicroscopy. Following full dilation of the pupil, all eyes underwent fundus examination through indirect ophthalmoscopy and photography (Daytona, Optos, United Kingdom). DR and other types of chorioretinopathy were diagnosed by two experienced licensed doctors based on the results of fundus examinations and assessments of all retinas images. Glycosylated hemoglobin A1c (HbA1c) and fasting blood glucose (FBG) concentrations were measured in all patients diagnosed with DR and DM.

### 2.4 OCT and OCTA data

All subjects underwent SS-OCT and SS-OCTA (TowardPi BMizar, TowardPi Medical Technology, Beijing, China) examinations while their pupils were still dilated. This newly developed SS-OCT/SS-OCTA system has an A-scan rate of 400 KHz, an axial scan depth of 6 mm, a B-scan length of 24 mm, and OCTA area of 24 × 20 mm. This system has an axial optical resolution of 3.8-µm and a transverse resolution of 10-µm, enabling the capture of high-resolution fundus images. The ability of this system to acquire OCT images 24-mm in width and 6-mm in depth, as well as ultrawide OCTA images of 24 × 20 mm, enabled the determination of information on chorioretinal vascular density (VD) and thickness at 24 × 20 mm in a single capture. Images were subsequently graded 1–10 by the SS-OCTA platform, with only images graded ≥8 utilized in the present study.

**Figure 2:**
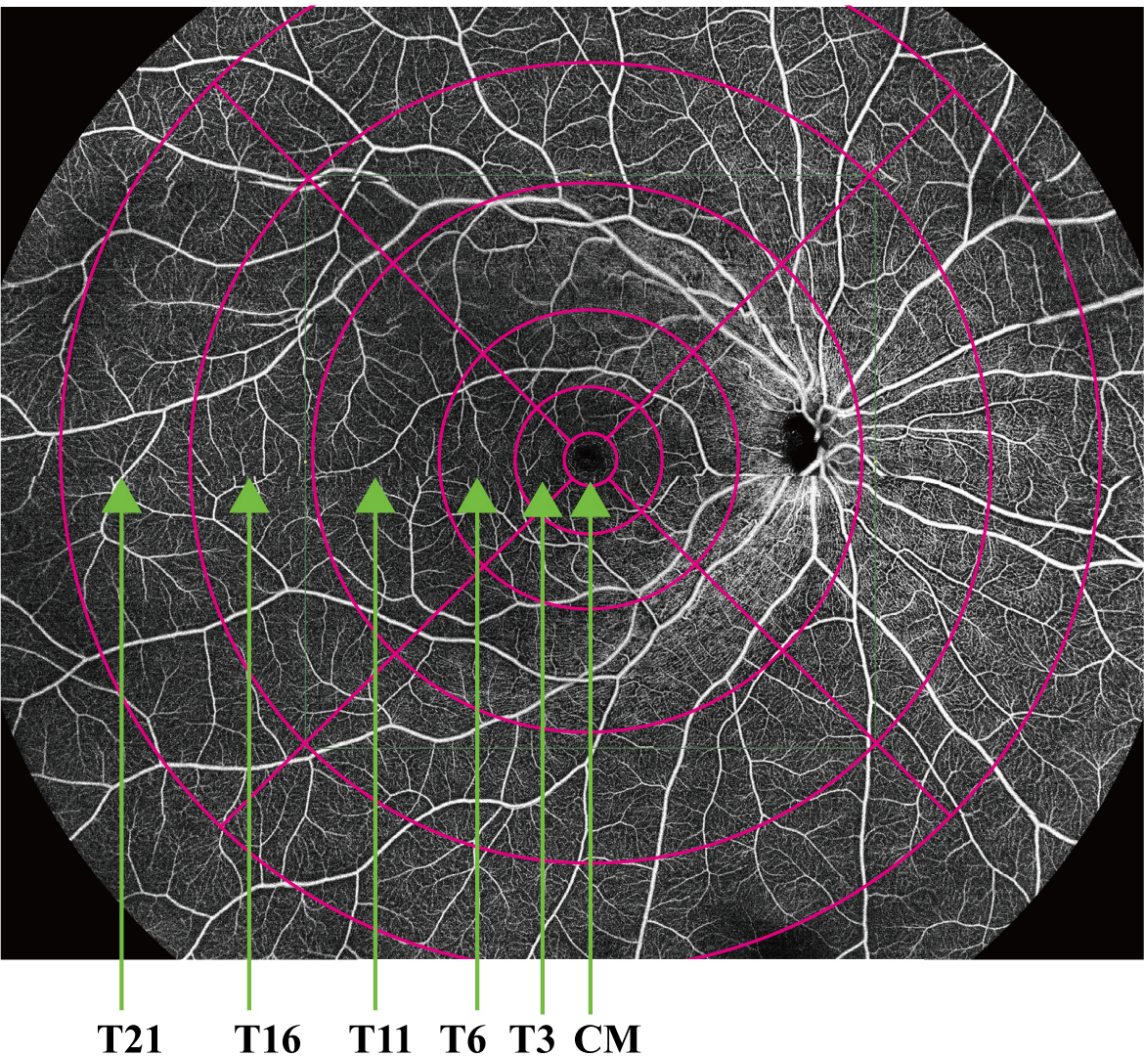
Vascular density and thickness of the central macula (CM) (1 mm diameter) and temporal fan-shaped areas of 1–3 mm (T3), 3–6 mm (T6), 6–11 mm (T11), 11–16 mm (T16), and 16–21 mm (T21).

**Figure 3:**
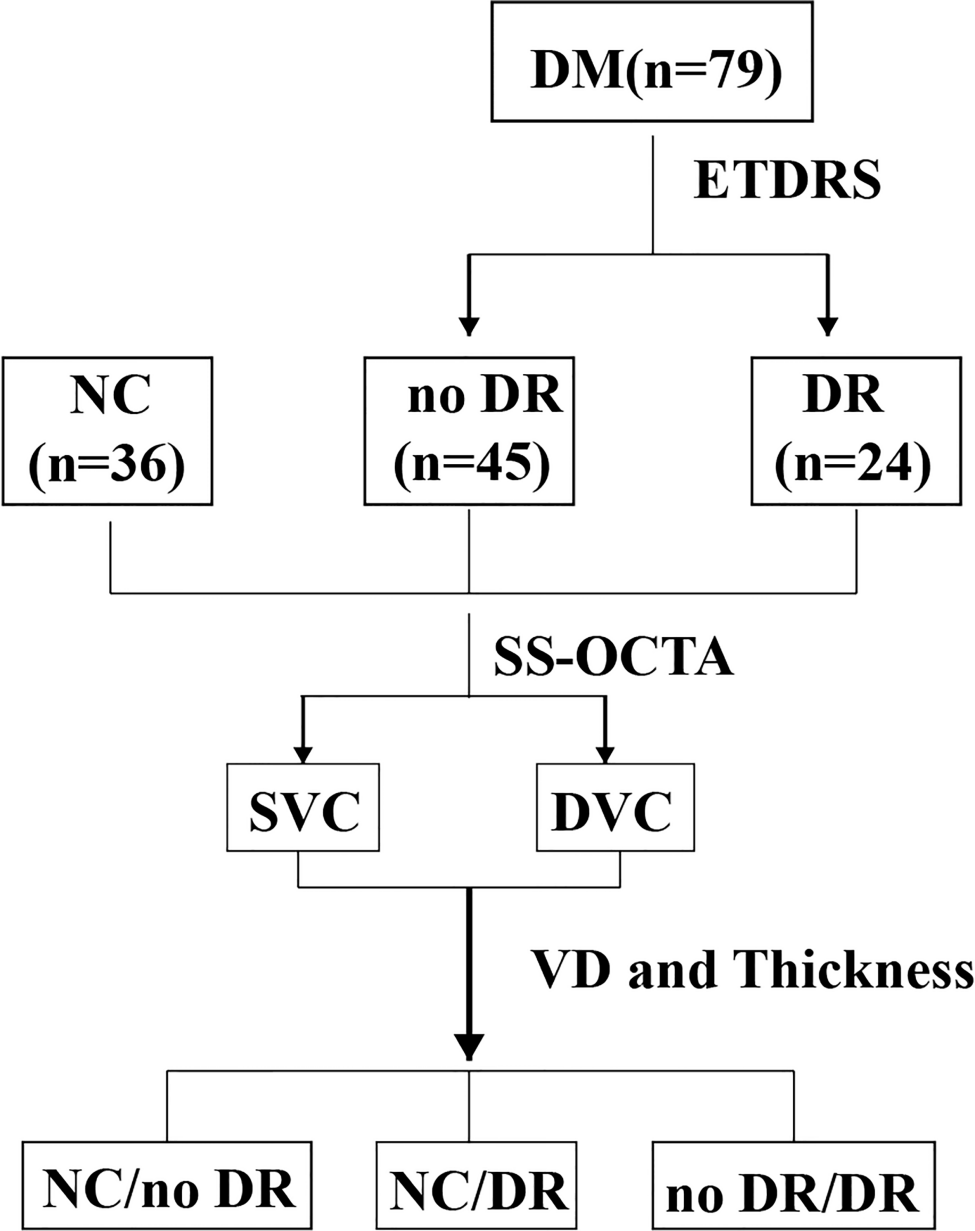
Flow diagram of the experimental design. DM, diabetes mellitus; ETDRS, Early Treatment Diabetic Retinopathy study; NC, normal control; DR, diabetic retinopathy; SS-OCTA, swept-source optic coherence tomography angiography; SVC, superficial vascular complex; DVC, deep vascular complex; VD, vascular density.

All the primary data were acquired and exported by the built-in software of the SS-OCT and SS-OCTA platform. The built-in Early Treatment Diabetic Retinopathy Study rings of the platform were applied to compare the different areas of the retina. The VD and thickness of the central macula (CM) (1 mm diameter) and of the temporal fan-shaped areas at 1–3 mm (T3), 3–6 mm (T6), 6–11 mm (T11), 11–16 mm (T16), and 16–21 mm (T21) (**Figure 2**) were compared in the DR, DM, and NC groups, as were the deep vascular complex (DVC), superficial vascular complex (SVC), and average thicknesses of the SVC and DVC nourishing segments. A flow diagram of the experimental design is shown in **Figure 3**.

### 2.5 Statistical analysis

All statistical analyses were performed using IBM SPSS software (version 26.0). The normal distribution of data was assessed using the Kolmogorov–Smirnov test. Data are presented as the mean ± standard deviation. Differences in age, right or left eye, AL, and other characteristics were analyzed by independent sample t-tests or analysis of variance (ANOVA). Differences in gender were evaluated by crosstabs analysis. Pairwise correlations among VD, thickness, and DR were analyzed by receiver operating characteristic (ROC) curve analysis. Areas under the ROC curve (AUC) >0.8 were considered significant. A p-value < 0.05 was regarded as statistically significant.

## 3 Results

### 3.1 Baseline data

This study enrolled 47 eyes from 24 patients with, 87 eyes from 45 patients with DM without DR, and 71 eyes of 36 age- and gender-matched control subjects. The baseline demographic and clinical characteristics of these three groups are shown in **Table 1**. The three groups showed no significant differences in numbers of subjects (p = 0.181), age (p = 0.083), FBG (0.059), AL (p = 0.087), and Hypertension (p = 0.859).

**Table 1:** Baseline demographic and clinical characteristics of DR patients, DM patients and healthy controls.

	Normal Control	DM	DR	P Values
Patients (Female)	36 (23)	45 (20)	24 (8)	0.181
Age, yr	54.00 ± 9.83	58.20 ± 10.22	53.88 ± 7.96	0.083
Eyes	71	87	47	0.999
Type of DM	N/A	2	2	N/A
Duration of DM, yr	N/A	7.02 ± 6.17	11.38 ± 7.10	0.010
FBG, mmol/L	N/A	7.82 ± 2.36	9.24 ± 3.45	0.059
HbA1c, %	N/A	7.42 ± 1.48	9.12 ± 2.09	0.000
AL, mm	23.33 ± 0.69	23.60 ± 0.85	23.51 ± 0.79	0.087
Hypertension	36 (10)	45 (14)	24 (6)	0.859

Values are shown as means ± SD. A P value less than 0.05 was considered statistically significant.

DM, diabetes mellitus; DR, diabetic retinopathy; FBG, fasting blood-glucose; HbA1c, hemoglobin A1C; AL, axial lengths; N/A, not applicable.

### 3.2 VD and thickness analysis of the SVC and DVC

**Figure 4:**
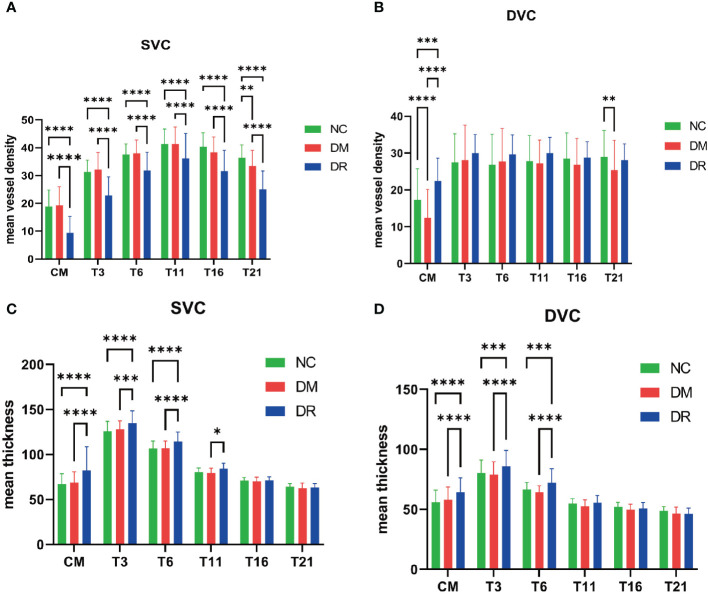
Vascular density (VD) and thickness analysis of the superficial vascular complex (SVC) and deep vascular complex (DVC). **(A)** Compared with the normal control (NC) group, **the** average VDs of the SVC were significantly lower in all retinal areas of patients with diabetic retinopathy (DR), as well as being significantly lower in the temporal fan-shaped area of 16–21 mm (T21) in patients with diabetes mellitus (DM). **(B)** The average VDs of the DVC in the central macula (CM) and T21 were lower in DM patients; although the average VDs of the DVC in all areas were higher in DR patients, only the changes in the CM were statistically significant. **(C)** The average thicknesses of SVC nourishing segments did not differ significantly in the NC and DM groups. The average thicknesses of these segments in the CM and temporal fan-shaped areas of 1–3 mm (T3), 3–6 mm (T6), and 6–11 mm (T11) were significantly greater in the DR than in the DM group. **(D)** The average thicknesses of DVC nourishing segments did not differ significantly in the NC and DM groups. The average thicknesses of these segments in the CM, T3, and T6 were significantly greater in the DR than in the DM group. *p < 0.05, **p < 0.01, ***p < 0.001, and ****p < 0.0001.

The average VDs of the SVC in the CM, T3, T6, T11, T16, and T21 areas were significantly lower in the DR than in the NC and DM groups, whereas only the average VD of SVC in the T21 area was significantly lower in the DM than in the NC group (**Figure 4A**). The average VDs of the DVC in the CM and T21 area were significantly lower in the DM than in the NC group (**Figure 4B**). Compared with the DM group, the average VDs of the DVC in the CM and T3, T6, T11, T16, and T21 areas were higher in the DR group, but only the CM difference was statistically significant (**Figure 4B**). The average thicknesses of SVC nourishing segments in the CM and in the T3, T6, and T11 areas were significantly higher in the DR than in the DM group, whereas the average thicknesses of SVC nourishing segments in the T16 and T21 areas did not differ significantly in the three groups (**Figure 4C**). The average thicknesses of DVC nourishing segments in the CM and the T3, and T6 areas were significantly higher in the DR than in the DM group, whereas the average thicknesses of DVC nourishing segments in the T11, T16 and T21 areas did not differ significantly in the three groups (**Figure 4D**). The average thicknesses of SVC and DVC nourishing segments did not differ significantly in the DM and NC groups (**Figures 4C, D**). All of these findings are summarized in **Table 2**.

**Table 2:** Comparisons of superficial and deep complex vessel densities and thickness among the NC, DM, and DR groups data.

		NC	DM	DR	p1 (NC vs DM)	p2 (NC vs DR)	p3 (DM vs DR)
Superficial Vascular Complex Vessel Densities	CM	18.90 ± 5.92	19.33 ± 6.66	9.40 ± 5.89	0.8907	<0.0001	<0.0001
	T3	31.31 ± 4.20	32.13 ± 6.12	22.85 ± 6.70	0.6578	<0.0001	<0.0001
	T6	37.54 ± 3.82	37.97 ± 4.80	31.83 ± 6.58	0.8895	<0.0001	<0.0001
	T11	41.25 ± 5.49	41.25 ± 6.17	36.15 ± 8.97	0.9999	<0.0001	<0.0001
	T16	40.34 ± 5.01	38.30 ± 5.55	31.60 ± 7.42	0.0739	<0.0001	<0.0001
	T21	36.41 ± 4.62	33.45 ± 5.59	25.02 ± 6.61	0.0046	<0.0001	<0.0001
Deep Vascular Complex Vessel Densities	CM	17.27 ± 8.52	12.43 ± 7.74	22.43 ± 6.18	<0.0001	0.0005	<0.0001
	T3	27.49 ± 7.74	28.13 ± 9.46	29.96 ± 5.09	0.8495	0.17	0.3449
	T6	26.89 ± 8.24	27.72 ± 8.97	29.66 ± 5.26	0.7556	0.1067	0.3021
	T11	27.86 ± 6.93	27.22 ± 6.34	30.00 ± 4.27	0.8445	0.2619	0.0869
	T16	28.55 ± 6.94	26.85 ± 7.09	28.83 ± 4.27	0.3102	0.9771	0.2897
	T21	28.94 ± 7.17	25.42 ± 8.02	28.11 ± 4.37	0.007	0.8139	0.1028
Superficial Vessel Complex Thicknesses	CM	67.11 ± 11.52	68.9 ± 11.78	82.19 ± 26.38	0.4619	<0.0001	<0.0001
	T3	125.76 ± 11.09	128.1 ± 9.33	134.74 ± 13.77	0.2647	<0.0001	0.0003
	T6	106.54 ± 8.34	106.86 ± 8.11	114.38 ± 10.53	0.9743	<0.0001	<0.0001
	T11	80.55 ± 4.43	79.37 ± 5.41	84.28 ± 6.01	0.7121	0.0888	0.0112
	T16	70.92 ± 3.52	70.06 ± 4.91	71.34 ± 3.84	0.836	0.9687	0.7316
	T21	64.23 ± 3.32	62.57 ± 5.71	63.34 ± 4.19	0.516	0.8712	0.8946
Deep Vessel Complex Thicknesses	CM	55.9 ± 10.12	58 ± 10.68	64.34 ± 11.85	0.2138	<0.0001	<0.0001
	T3	80.28 ± 10.82	78.85 ± 10.85	85.96 ± 13.19	0.4868	0.0003	<0.0001
	T6	66.54 ± 5.94	64.34 ± 5.39	72.32 ± 11.57	0.1865	0.0003	<0.0001
	T11	54.93 ± 4.04	52.68 ± 5.29	55.55 ± 5.97	0.1697	0.9056	0.1051
	T16	52.1 ± 3.74	49.64 ± 4.53	50.85 ± 4.86	0.1218	0.6728	0.6699
	T21	48.83 ± 3.45	46.6 ± 5.16	46.3 ± 4.76	0.1746	0.1969	0.9756

All results are shown as means ± SD.

NC, normal control; DM, diabetes mellitus; DR, diabetic retinopathy.

### 3.3 ROC curves of VD in the DM and DR groups

**Figure 5:**
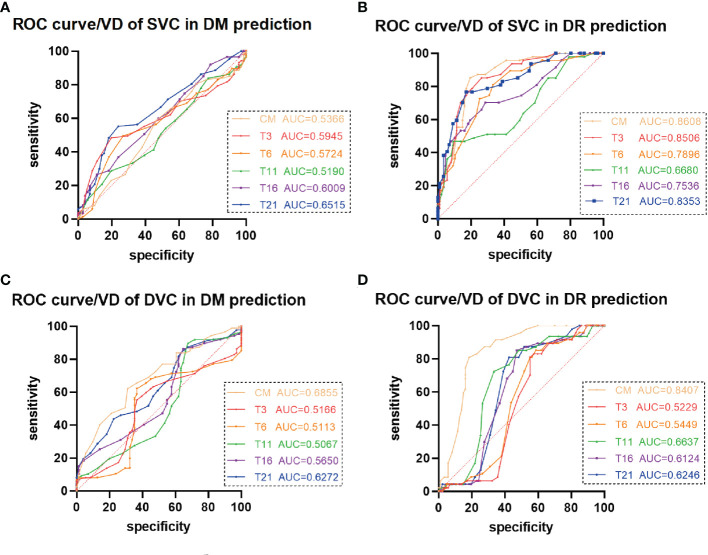
Receiver operating characteristic (ROC) curve analysis of vascular density (VD) in patients with diabetes mellitus (DM) and diabetic retinopathy (DR). **(A**, **C)** The average VDs of the **(A)** superficial vascular complex (SVC) and **(C)** deep vascular complex (DVC) showed poor ability to predict DM. **(B)** The average VDs of the SVC in the central macula (CM) and temporal fan-shaped areas of 1–3 mm (T3) and 16–21 mm (T21) revealed better ability to predict DR. **(D)** The average VD of the DVC in the CM revealed ability to predict DR.

ROC curve analysis of the average VDs of the SVC showed that the AUCs were 0.8608 (95% CI 0.7982–0.9235, p < 0.0001) for the CM, 0.8506 (95% CI 0.7852–0.9159, p < 0.0001) for the T3 area, and 0.8353 (95% CI 0.7641–0.9065, p < 0.0001) for the T21 area, showing that these parameters were predictive of DR development (**Figure 5B**). The optimal CM cut-off value predicting DR was 15.50, with a sensitivity of 85.11%, a specificity of 80.46%, and a Youden Index of 0.66. The optimal T3 cut-off value predicting DR was 29.50, with a sensitivity of 85.11%, a specificity of 73.56%, and a Youden Index of 0.59. The optimal T21 cut-off value predicting DR was 28.50, with a sensitivity of 76.60%, a specificity of 82.76%, and a Youden Index of 0.59. In the DVC, only the average VD in the CM was predictive of DR (**Figure 5D**), with an AUC of 0.8407 (95% CI 0.7735–0.9078, p < 0.0001). The optimal CM cut-off value predicting DR was 17.50, with a sensitivity of 80.85%, a specificity of 81.61%, and a Youden Index was 0.62. The average VDs of the SVC and DVC were not predictive of DM (**Figures 5A, C**), and the average thicknesses of the SVC and DVC were not predictive of either DM or DR (**Supplementary Figure 1**).

## 4 Discussion

This cross-sectional, observational study analyzed the average VD of the SVC and DVC and the average thicknesses of SVC and DVC nourishing segments in patients among the NC, DM, and DR groups. Using the newly developed SS-OCT and SS-OCTA system, this study analyzed changes to the peripheral retina (21 mm) in the three groups. The average VDs of the SVC in all observed areas were significantly lower in the DR than in the NC group, whereas only the T21 area showed a significant decrease in the DM compared with the NC group. The average VDs of the DVC in the CM and T21 area were significantly lower in the DM than in the NC group. No significant changes in the thicknesses of SVC or DVC nourishing segments were observed in the peripheral retina (21 mm). ROC curve analysis showed that the average VDs of the SVC in the CM and in the T3 and T21 areas revealed were predictive of DR development, whereas only the average VD of the DVC in the CM was predictive of DR.

Recent developments in ultrawide OCT and OCTA equipment has resulted in the ability to image areas measuring 24 × 20 mm, the maximum range currently available among all OCTA devices (18, 19). Use of this advanced equipment has shown that the density of the choroidal vessels and the thicknesses of the inner retina segments at the periphery were significantly lower in nonpathological myopic fundi (16). This equipment was also found to clearly delineate choroidal osteomas from the adjacent vessels in the choroidal Sattler’s and Haller’s layers (16), providing further evidence that the newly developed OCT and OCTA system can simultaneously acquire ultrawide and high-resolution images of the fundus. At present, 3 × 3 mm and 6 × 6 mm OCT and OCTA devices are widely used in clinical practice, with the use of 12 × 12 mm devices increasing, providing additional information about the peripheral retina. Using these 12 × 12 mm OCT and OCTA devices, retinal microvascular abnormalities have been observed in the peripheral retinas of DM patients without DR (15), with the 24 × 20 mm devices providing additional information on the peripheral retina.

Fundus fluorescein angiography (FFA) is a traditional imaging technique for DR, as it can show retinal neovascular and vascular leakage. This method, however, is invasive and expensive, carries a risk of allergy, and can negatively affect the liver and kidneys, thus limiting its widespread use (20, 21). SS-OCTA may be a good alternative, as it can avoid these problems. Although a comparison of 24×20 mm^2^ SS-OCTA with FFA in evaluating DR lesions showed good to moderate agreement, SS-OCTA had several advantages, including its noninvasiveness, lower cost, rapid performance, and reproducible results (22). A recent study analyzing the effect of scan area on the detection of DR lesions, by comparing 12 mm × 12 mm central and 24 mm × 20 mm images, found that the ultra-widefield SS-OCTA detected more intra-retinal microvascular and neovascular abnormalities, with higher rates of ischemia in the mid-peripheral than in the posterior retina (23). Another study found that ultra-widefield color fundus photography (UWF CFP) plus SS-OCTA and UWF CFP plus FFA showed good agreement in the rate of detection of DR lesions and DR severity grade (24). Taken together, these findings suggested that ultra-wide SS-OCTA has advantages when compared with FFA or 12 mm × 12 mm OCTA, with the potential to be a rapid, repeatable and noninvasive method for DR screening and follow-up.

Studies using 3 × 3 mm OCT and OCTA devices have found no significant differences in the VD and thickness of the superficial and deep retinal layers between DM and NC groups (25–27), findings partially consistent with the results of the present study. In contrast, other studies have found that the VD and thickness in the parafoveal and/or perifoveal layers are significantly lower in patients with DM than in NCs (28–30). These studies differ from the present study in their inclusion and exclusion criteria. Most previous studies defined DM without DR based on fundus photography, whereas the present study excluded DR based on both 24 × 20 mm OCTA and fundus photography. Microaneurysms and fovea avascular zone changes can be detected by OCTA in DM patients through normal fundus photography (28, 31). These subjects were excluded from the present study based on the results of high-resolution and ultrawide field OCTA.

The present study showed early VD changes of the SVC and DVC in the peripheral retina (21 mm) in patients with DM, as well as in patients with DR. ROC curve analysis found that the VD of the SVC in T21 showed good ability to predict DR. Moreover, the VD of the SVC in T21 was the only significant change in the SVC in patients with DM. These results indicate that the VD of T21 is an early indicator of DR progression and may be a biomarker predictive of DR. The VD of the SVC in all areas was significantly lower in patients with DR, a finding consistent with previous results (15, 29, 32). The newly developed OCTA device expanded the detectable area to T16 and T21, thereby consolidating previous results. The findings in patients with DM also suggested that retinal circulation was affected before clinical manifestations of DR (28, 33). The decreased VD in the SVC in DR results largely from an incremental loss of capillary segments (34), whereas the increased VD in the DVC is regarded as an autoregulatory response to increased metabolic demand in mild to moderate DR. The present study found that SVC nourishing segments in the CM, T3, T6, and T11 and DVC nourishing segments in the CM, T3, and T6 were thicker in patients with DR, a finding consistent with a previous study using another type of OCTA device (35).

The present study had several limitations. First, demographic information, DM duration, and other previous medical history was self-reported by the study subjects. Collecting more data can help avoid potential bias in relation to the duration of diabetes and blood glucose control. Studies using a larger number of patients, as well as measurements of blood pressure, lipids, and other laboratory data, are required to confirm the present findings. Studies should also include patients with progressive DR, thus allowing longitudinal determinations of changes in retinal structure during the progression of DR.

In conclusion, the present study showed that the progression of DR was accompanied by changes in the peripheral retina. The ultrawide and rapid scanning SS-OCT and SS-OCTA system expanded the detectable area to 24 × 20 mm. Prospective studies including larger numbers of patients are needed to confirm these findings and expand the use of the newly developed SS-OCT and SS-OCTA device.

## Data availability statement

The raw data supporting the conclusions of this article will be made available by the authors, without undue reservation.

## Ethics statement

The studies involving human participants were reviewed and approved by the Ethics Committee of Sichuan Provincial People’s Hospital. The patients/participants provided their written informed consent to participate in this study.

## Author contributions

JZ, JL, WD, YZ, ML—involved in study conceptualization. MyL, DW, MM, XL—involved in study supervision. ML, SC, YL, BC, LY, LS, LQ, RZ—involved in data acquisition and data analysis. JZ, YZ—involved in data interpretation; YZ, ML—involved in the drafting of the manuscript. JZ, JL, WD, ZY, ML—involved in interpretation of results. JZ, JL, WD, ZY, ML—involved in critical review of the manuscript. All authors contributed to the article and approved the submitted version.
