# Supplementary figures and images for: Early changes to retinal structure in patients with diabetic retinopathy as determined by ultrawide swept-source optical coherence tomography-angiography
Source: Front Endocrinol (Lausanne). 2023 May 8;14:1143535. doi: 10.3389/fendo.2023.1143535 (PMC10200911; doi:10.3389/fendo.2023.1143535)

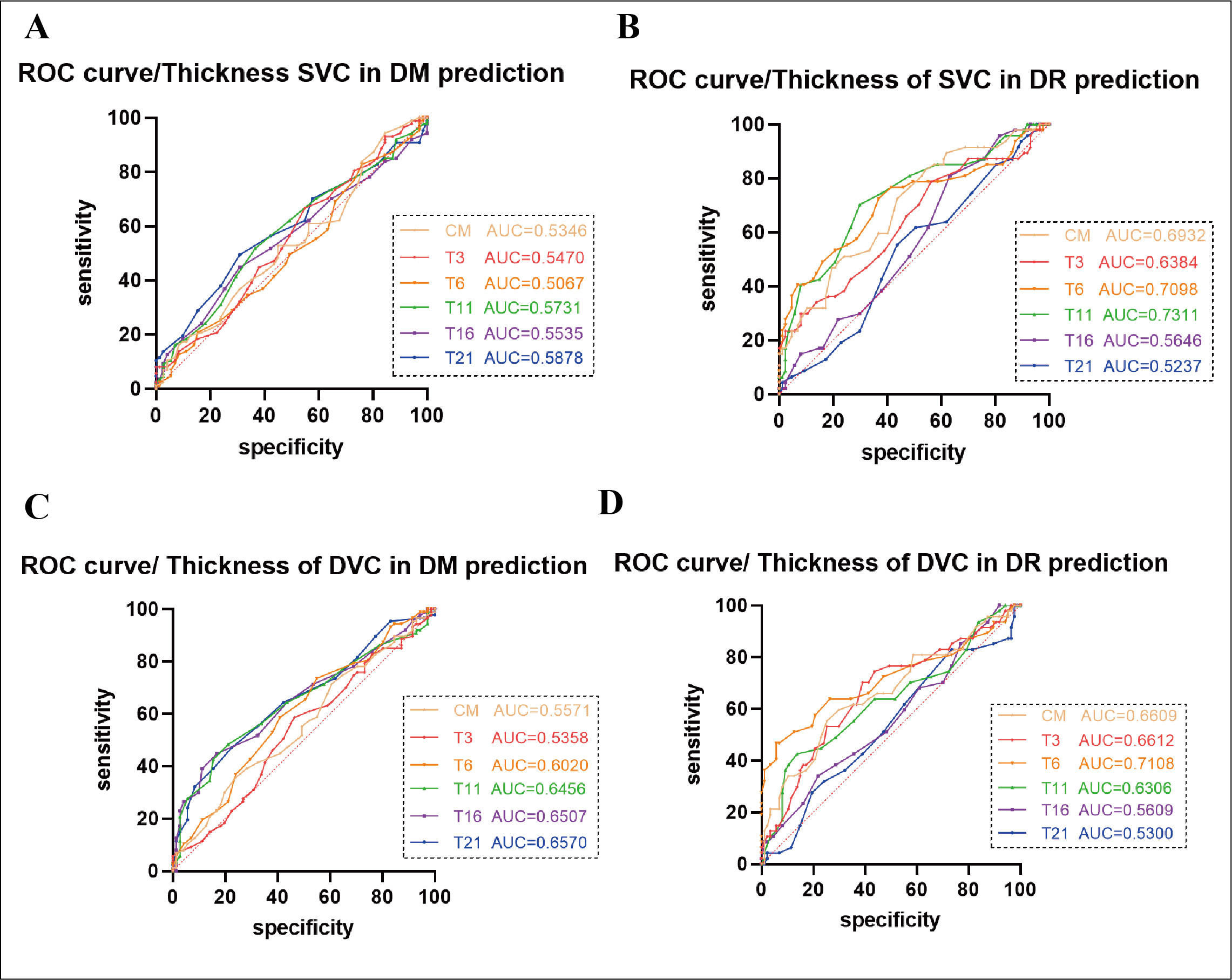

Supplement: Supplementary file 1 [file Image_1.jpeg]
